# Rare Case of Pediatric Upper Airway Obstruction: Laryngeal Granular Cell Tumor

**DOI:** 10.7759/cureus.22829

**Published:** 2022-03-03

**Authors:** Marcos Mirambeaux, Henry Paulino, Ricardo Acra-Tolari, Michelle Matos, Carlos Esteva

**Affiliations:** 1 Otolaryngology - Head and Neck Surgery, General Hospital Plaza de la Salud, Santo Domingo, DOM; 2 Otolaryngology - Head and Neck Surgery, General Hospital Plaza de la Salud, Santo Domingo , DOM; 3 Pathology, ESTEVA Patología Diagnóstica, Santo Domingo, DOM

**Keywords:** flexible laryngoscopy, granular cell tumor, upper airway, dysphonia, laryngeal

## Abstract

Granular cell tumors (GCTs) are uncommon neoplasms of unknown origin that can manifest in multiple locations throughout the body. Physicians should be aware of this type of tumor presenting in unusual locations such as the larynx, particularly in pediatric patients with stridor and dysphonia. We describe an 11-year-old female with a large laryngeal mass that obstructed the majority of the laryngeal lumen. A tracheotomy was performed to secure the patient's airway, followed by a direct suspension laryngoscopy, during which the mass was excised in its entirety. The biopsied mass was histopathologically and immunohistochemically examined to confirm the diagnosis of granular cell tumor. She benefited from treatment and experienced a favorable outcome. This case report emphasizes the critical nature of properly diagnosing this type of tumor in patients who present with vocal or respiratory symptoms.

## Introduction

Granular cell tumors (GCTs) are uncommon, usually benign, and of controversial origin. Abrikossoff first described them as myogenous tumors in 1926. Recent evidence, however, indicates an immunohistochemical and electron microscopy association with Schwann cells, implying a neurogenic origin [1,2]. These tumors can arise in any part of the body, but 50% of cases occur in the head and neck region, with the tongue being the most common location. Around 3-10% of all cases occur in the larynx [2,3].

Granular cell tumors are most frequently encountered in patients in their third to sixth decades of life and are uncommon in children. There appears to be a higher incidence among African-Americans and females, although laryngeal involvement is more prevalent in males [2-4]. Exact world statistics are not adequately established and most of the literature, including the laryngeal presentation of granular cell tumors, consists of a few case reports. This article, being the first case of a GCT in the larynx reported in the Dominican Republic, focuses on the patient's presentation, diagnosis, and management.

## Case presentation

An 11-year-old Dominican female presented to our clinic with her mother, who described a one-year history of progressive dysphonia, vocal fatigue, and respiratory distress during sleep, which were associated with episodes of apnea. The patient's symptoms had been deteriorating for six months, prompting them to seek treatment.

During the initial consultation, a flexible laryngoscopy revealed a large, heterogeneous mass obstructing the majority of the laryngeal lumen. The remainder of the examination was uneventful, and hospitalization was warranted.

**Figure 1:**
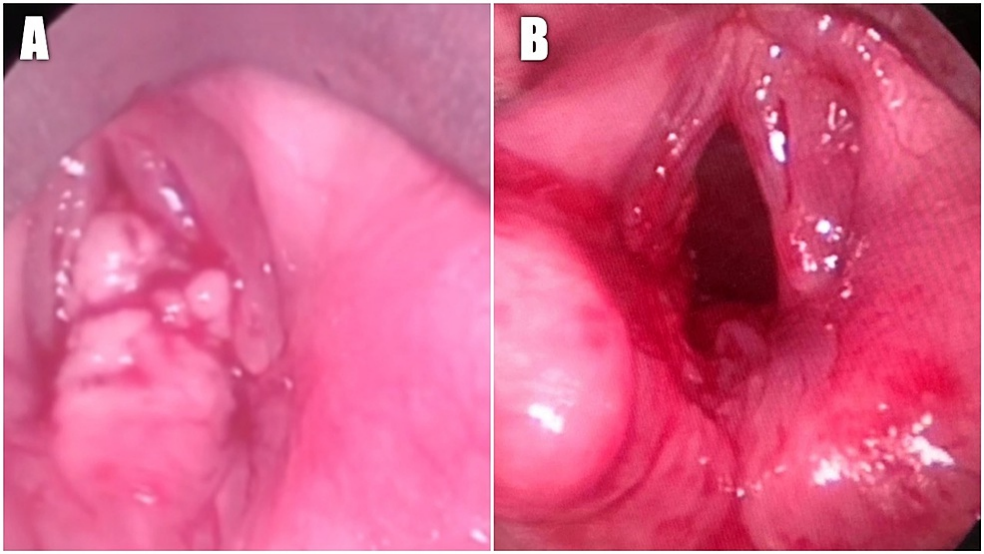
Intra-operative endoscopic visualization of the larynx. (A) Visualization of a large, heterogeneous lesion arising from the posterior larynx and (B) larynx after mass resection.

The same day, the patient was taken to the operating room, where a tracheostomy was performed to secure the patient’s airway, followed by a direct suspension laryngoscopy, which visualized a heterogeneous lesion with an indurated regular base and a distal exophytic, irregular and solid component, that emerged from the posterior commissure (Figure 1A). Due to these findings, wide endoscopic excision of the lesion was done (Figure 1B), leading to no complications.

**Figure 2:**
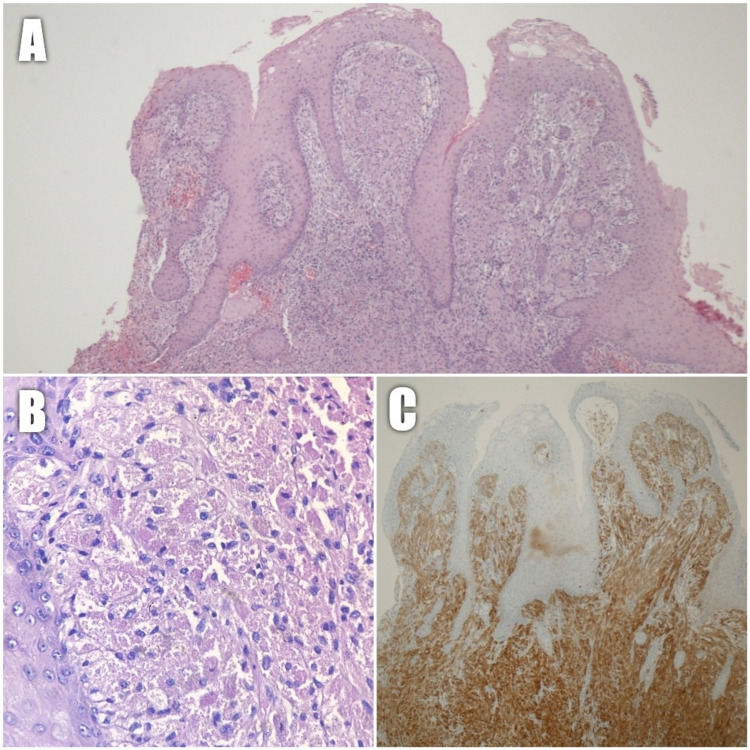
(A) Low power view of squamous mucosa with acanthosis, papillomatosis, and subepithelial epithelioid cell proliferation (H&E 100× magnification). (B) High power view of the epithelioid cell proliferation composed of large polygonal cells with abundant granular eosinophilic cytoplasm and small central nuclei (H&E 400× magnification). (C) S100 Protein immunostain is strongly positive in tumor cells. H&E: hematoxylin and eosin.

Polypoid fragments of squamous mucosa with acanthosis, papillomatosis, and subepithelial proliferation of epithelioid cells with abundant granular eosinophilic cytoplasm and a small nucleus were described in the histopathological report (Figure 2A-2B). These cells were immunohistochemically positive for Vimentin and S100 protein (Figure 2C). This confirmed neuronal differentiation and, in conjunction with the morphological findings, established the diagnosis of laryngeal granular cell tumor.

The patient experienced no complications during the immediate postoperative period. One week after the procedure, scar tissue was visualized in the left posterior larynx during a control laryngoscopy. The tracheostomy tube was removed. Subsequently, the patient's prior symptoms completely resolved.

The patient was scheduled for a general review two years after surgery. Flexible laryngoscopy revealed that both vocal cords appeared and functioned normally. No evidence of recurrence was observed, and vocal and respiratory symptoms remained absent.

## Discussion

Granular cell tumors of the larynx are uncommon in the pediatric population, where approximately fewer than 30 cases have been reported [5]. The etiology is not well established, but local laryngeal trauma, whether physical or chemical, as well as degenerative, regenerative, and congenital processes, have been proposed [1,6,7].

Clinical manifestations depend on the size of the lesion, presenting with chronic dysphonia and, less frequently, stridor, hemoptysis, dysphagia, otalgia, and dyspnea. GCTs of the larynx tend to be firm, sessile, small masses (less than 2 cm) covered by intact mucosa that surrounds the posterior third of the true vocal cords, but they have also been found in the anterior commissure, arytenoids, false vocal cords, subglottis, and postcricoid region [1,2,7].

Histopathology and immunohistochemistry examinations of the excised mass are used to make the diagnosis. Because 50-65% of cases develop pseudoepitheliomatous squamous hyperplasia on top of the tumor, a biopsy of the mucosa alone may result in the misdiagnosis of a well-differentiated squamous cell carcinoma [1,2].

GCTs cells are large and polygonal, with a granular appearance caused by an accumulation of densely packed cytoplasmic lysosomes [5]. These cells are immunohistochemically positive for the S-100 protein and neuron-specific enolase (NSE). Additionally, they can stain for Vimentin, Myelin-Associated Glycoprotein (Leu-7), and CD68 (KP-1), but not for smooth muscle actin (SMA), HMB45, Melan A, or Desmin [1].

The vast majority of these tumors are benign. Malignancy affects only 0.6 percent of the population [6]. Malignant tumors are typically larger than 4 cm in diameter, located in the skin and subcutaneous region, involving regional lymph nodes, growing rapidly, and exhibiting distant metastases. Malignant GCTs have a poor prognosis due to their high chemo-radioresistance. Death occurs approximately two years after diagnosis [5,6].

Recurrences have been described in up to 2% to 8% of cases. However, these may be new primary lesions as they can appear in several parts of the body in up to 10% of patients [8]. Due to the rarity of these tumors, there are no widely recognized guidelines for follow-up. However, the majority of authors recommend long-term surveillance due to the possibility of recurrences, metastases, and the development of new head and neck malignancies [5,8].

## Conclusions

GCTs of the larynx are a rare cause of upper airway obstruction. Physicians should be aware of this, especially in pediatric patients, where symptoms such as stridor and dysphonia open up a broad range of possibilities. Complete excision with wide negative margins is required for treatment. If appropriate management is provided, recurrence is uncommon, and the prognosis is favorable.

Our patient presented with a large mass, hence a higher complication risk. Nevertheless, she benefited from treatment and had an excellent outcome. Due to the limited number of case reports in the literature and the fact that this is the first case reported in our country, patients exhibiting these symptoms should be granted greater attention and considered for this diagnosis. This case report emphasizes the importance of accurately diagnosing this type of tumor, as they can pose a significant risk of upper airway compromise if not detected early.

## References

[1] Park JH, Do NY, Cho SI, Choi JY (2010). Granular cell tumor on larynx. Clin Exp Otorhinolaryngol.

[2] Mur TA, Pellegrini WR, Tracy LF, Levi JR (2020). Laryngeal granular cell tumors in children: a literature review. Int J Pediatr Otorhinolaryngol.

[3] Valldeperes A, Thomas-Arrizabalaga I, Alvarez-Ceballos L, Landa M (2020). Granular cell tumors of the larynx: a clinicopathologic study of five patients. J Voice.

[4] Lazar RH, Younis RT, Kluka EA, Joyner RE, Storgion S (1992). Granular cell tumor of the larynx: report of two pediatric cases. Ear Nose Throat J.

[5] Hogan C, Acharya V, Tsitsiou Y, Taghi A (2020). Laryngeal granular cell tumour: a very rare diagnosis for a child presenting with hoarse voice in the UK. BMJ Case Rep.

[6] Sataloff RT, Ressue JC, Portell M, Harris RM, Ossoff R, Merati AL, Zeitels S (2000). Granular cell tumors of the larynx. J Voice.

[7] Scala WR, Fernandes AF, Duprat AC, Costa HO (2008). Tumor de células granulares da laringe na infância: relato de caso. Rev Bras Otorrinolaringol.

[8] Hwang I, Hwang JE, Choi SH, Nam SY, Cho KJ (2007). Granular cell tumors of the larynx: report of three cases. Korean J Pathol.

